# TAVR, SAVR and MI-AVR. Good Things Come to Those Who Wait

**DOI:** 10.3390/jcm9113392

**Published:** 2020-10-23

**Authors:** Antonio Piperata, Marco Gemelli, Vjola Jorgji, Gino Gerosa, Tomaso Bottio

**Affiliations:** 1 Department of Cardiac, Thoracic, Vascular, and Public Health Sciences, University of Padua, via N. Giustiniani, 2, 35128 Padova, Italy; antonio.piperata@studenti.unipd.it (A.P.); marco.gemelli@studenti.unipd.it (M.G.); gino.gerosa@unipd.it (G.G.); 2 Hacohen Lab, Massachusetts General Hospital, Boston, MA 02114 USA; vjorgji@broadinstitute.org

Modern medicine uses a combination of advanced technology and established knowledge to reach its ultimate goal: healing patients while limiting risks and preventing disease. Nevertheless, the growing influence of the economy and capitalist markets on therapeutic decision-making is yet to be clarified. Consider, for example, the current debate around the treatment of aortic valve disease. Although surgical aortic valve replacement (SAVR) has been considered the standard of care in the past few decades, recent years have seen a substantial rise in the minimally invasive transcatheter aortic valve replacement (TAVR) technology, even in low-risk surgical patients. Several trials have compared the outcomes of the two competing therapies in different patient populations. The results of the Placement of Aortic Transcatheter Valves (PARTNER) 1 trial showed that Edwards’ TAVR was superior to the standard therapy in patients who could not undergo surgery, and non-inferior to SAVR in high risk patients in terms of death at 30 days and 1 year [1,2]. Similar results were obtained by the CoreValve Pivotal US trial, when investigating Medtronic TAVR in patients with high surgical risk [3,4]. For patients with intermediate surgical risk, Leon et al. [5] showed that TAVR was comparable to surgical AVR regarding the primary end point of death or disabling stroke. The evidence from the aforementioned trials was taken into account in 2017, when the American College of Cardiology/American Heart Association’s Guidelines [6] were updated to indicate TAVR as class I for high and prohibitive risk patients, and class IIA for those at intermediate risk.

Although these developments represented a huge step forward for TAVR utilization, the big leap was only taken in 2019. The Evolut Low Risk (LR) trial [7] showed non-inferiority of self-expanding TAVR over SAVR in terms of death or disabling stroke at two years in low surgical risk patients. Similarly, the PARTNER 3 trial [8] demonstrated the non-inferiority of balloon-expandable TAVR for the composite outcome of death, disabling stroke, or rehospitalization at one year. These two breakthrough clinical trials led to the US Food and Drug Administration (FDA) approving the use of TAVR for patients at low surgical risk, marking a huge shift in the treatment and management of aortic valve disease.

Despite its rising popularity, some literature has emerged to challenge the implementation of TAVR. A meta-analysis published by Barili et al. [9] found that, despite the significantly lower incidence of death in the first year after valve implantation in the TAVR group, TAVR was a risk factor for all-cause mortality after 40 months. Additionally, Kaul [10] raised questions about the bias of the Evolut LR and PARTNER 3 trials, concluding that longer follow-ups and a sufficient number of events will be required to justify a strong recommendation for TAVR in low-risk patients.

To sum up, TAVR has been proven to be more effective than the SAVR standard therapy in ineligible surgical candidates, as well as high-risk patients. There is some additional evidence, limited by the shortness of the follow-up, suggesting its non-inferiority in intermediate and low-risk patients. The advantages of TAVR in ineligible and high-risk patients are now recognized by the entire scientific community. What is not fully understood is the reason why great efforts have been made to broaden the indication of TAVR to low-risk patients, despite the uncertain proof of its long-term superiority over SAVR. In this context, a recent propensity score analysis at five years showed that the intermediate and low-risk patient groups treated with TAVR performed significantly worse than the SAVR group [11].

In attempting to explain the massive adoption of TAVR in recent years, it is worth characterizing the treatment as more of an advancement in technology rather than technique. As explained by D’Onofrio and Gerosa [12], technology is a dynamic and ever-evolving concept, whereas technique is static. Therefore, we expect that further breakthroughs in TAVR technology will heighten its performance to equal or exceed that of SAVR. However, this new technology is significantly more expensive than SAVR, while its actual benefit for some risk profiles remains controversial.

The substantially higher cost of TAVR has prompted several studies to evaluate its cost effectiveness. A systematic review [13] revealed that TAVR may be medically and economically justified, if compared to medical therapy, for unsuitable surgical candidates. However, there is insufficient evidence that economically justifies TAVR over SAVR in intermediate and low-risk patients. Similar reports [14,15,16] demonstrated that, in Europe, TAVR is not a cost-effective strategy for intermediate and high-risk patients. Importantly, TAVR has been found to cost up to twice as much as SAVR, despite not significantly reducing the mortality rate [17]. In this context, Ailawadi et al. note that “when a new technology costs more but delivers outcomes inferior to those observed with established technology, the new technology becomes a dominant technology rather than a more cost-effective approach” [17]. This situation creates a vicious cycle in which large companies are prone to invest more in products that bring in more revenue, thus growing the transcatheter technology market, while the development of surgical strategies is overlooked. This outcome defines the real risk we are facing: dominant products may prevail on therapeutic decisions. We firmly believe that medicine must not lose its ultimate goal of healing patients. History has taught us that every disease and patient is unique. For this reason, the therapeutic choices must be customized for each specific clinical case.

Moreover, in the lively debate about the treatment of aortic valve disease, the existence of minimally invasive AVR (MI-AVR) is often overlooked. In the panorama of cardiac surgery, MI-AVR has been steadily gaining widespread popularity due its undisputed advantages of less invasiveness and trauma, faster recovery, and overall improvement in the patients’ quality of life [18,19,20,21]. Furthermore, it is associated with reduced transfusion incidence and lower intensive care stay, hospitalization, and renal failure, compared to conventional AVR [18,19,20]. In addition, the MI-AVR approach, when associated with the use of modern sutureless (SU) and rapid-deployment (RD) prostheses, achieves excellent haemodynamic results with reduced cardiopulmonary bypass and cross-clamp times [21,22]. The combination of these features possibly makes MI-AVR the best compromise between technique, technology, costs, the reduction of complications and improved clinical results for some groups of patients.

This provocative vision allows us to emphasize that TAVR, SAVR and MI-AVR are all good and safe therapeutic options that represent a great achievement for modern medicine. To achieve the goal of personalizing care and therapies for individual patients, we believe that TAVR, SAVR and MI-AVR are all tools needed in the bag of the next generation of cardiac surgeons.
